# Study on emergency escape route planning under fire accidents in the Burtai coal mine

**DOI:** 10.1038/s41598-022-15437-7

**Published:** 2022-07-29

**Authors:** Jia Jinzhang, Wang Fengxiao

**Affiliations:** 1 College of Safety Science and Engineering, Liaoning Technical University, Fuxin, 123000 Liaoning China; 2 Key Laboratory of Mine Thermal Power Disaster and Prevention, Ministry of Education, Fuxin, 123000 Liaoning China

**Keywords:** Risk factors, Engineering

## Abstract

Developing an effective safety emergency plan for coal mines is crucial to prevent and reduce accidents, as well as improve the emergency response capability. This paper identifies and analyzes potential accident hazards in the Burtai coal mine through a comprehensive and practical investigation of risk factors. Based on a mathematical model of a mine fire disaster relief algorithm, the MATLAB software is used to conduct numerical simulations of the dynamic spreading process of the fire smoke. Several escape routes are determined based on the simulation results, including the main escape route at the working face of coal mining, the main escape route at the working face of tunneling, and the main escape route against the reverse wind in the main inclined shaft, the auxiliary adit, and the main air intake roadway. The results presented in this study can provide guidance for improving fire emergency escape and rescue plans of the Burtai coal mine.

## Introduction

The coal industry is a top high-risk industry with poor production conditions and a constantly changing operating environment. Many types of disasters and hazards such as floods, fires, gas, coal dust, and roof accidents can cause severe threats to the production safety of coal mines. Coal mine safety issues are the top priority in the national production safety^1–4^. Therefore, establishing an effective safety emergency plan is important for providing an appropriate response to an accident, escape, and rescue. When an accident occurs, providing a fast, orderly, and effective escape and rescue plan is necessary to ensure a rapid control of the accident and timely achieve self-rescue, mutual rescue, and disaster escape so as to minimize casualties and property losses^5–9^.

Underground mining is the main method of coal extraction in China and many other coal-producing countries. In recent years, with a continuous increase in mining depth, the possibility of various disasters, such as gas explosions, eruptions, gas outbursts, and mine floods, has increased^10,11^. When a major disaster occurs in a mine, it can severely hurt underground workers and even threaten their lives^12,13^. Fire, vehicle collisions, and rockfalls are the most common emergencies in mineral and metal underground mines^14,15^. In most areas of a mine, fire can affect roof stability and produce toxic gases, thus causing hypoxic conditions and making spontaneous escape and rescue operations impossible^16,17^. Mine rescue operations take a certain time regardless they are carried out by mine rescue teams or local rescue services and emergency medical services. There are time delays because the rescue team has to go into remote mines, arrange rescue operations, and navigate in complex environments to reach the accident site, which can be far underground. When a disaster happens, a large amount of toxic gases is generated, and the presence of fire and wind pressure can even accelerate gas diffusion. Therefore, workers must be evacuated rapidly from a disaster site to the safe zone. However, underground mines have complex structures with limited escape routes. These factors impose significant difficulties to escape planning and realization. Therefore, to reduce the death rate from accidents and ensure worker safety, it is critical to plan an escape route ahead^18–23^.

The Mine Safety Technology and Training Committee has recommended that mine workers must possess the following qualifications to ensure effective escape and rescue in the case of emergency: escape knowledge, ability to determine an evacuation route for a specific mine, and general knowledge about escape and rescue for decision making in emergency^24,25^. A lack of these qualifications can severely affect mining safety. At the very first moment of an emergency, understanding the current situation can significantly improve escape and rescue. A rescue route refers to an optimal route for the rescue personnel to enter the disaster area from a safe area, whereas an escape route refers to an evacuation route for the underground personnel to leave the dangerous area^26,27^.

At present, most researches on the planning of emergency escape routes are mostly carried out with indoor buildings as the research object. The geological structure of the mine is complex and the tunnels are crisscross, so the existing indoor disaster escape route research conclusion has little application value in the coal mine. The existing research on coal mine emergency escape route planning is mostly carried out from the existing safety management regulations. Through continuous improvement of emergency management system and continuous safety training, it aims to enable underground workers to master more disaster escape and escape skills as much as possible, so as to improve the success rate of emergency disaster escape. Every mine and every emergency situation are unique, and the causes of different coal mine accidents are different. For different mines, a certain disaster may lead to different consequences. Therefore, the research on emergency disaster escape route of mine fire should be carried out for a specific mine. The research results obtained in this way are universal and valuable.

In this work, we analyzed potential accidents of the Burtai coal mine and establishes a mathematical model to simulate the dynamic fire propagation process of a smoke flow. Based on the simulation results of the constructed model, optimal escape routes are determined. The results obtained from this work can provide guidance for fire emergency planning of the Burtai coal mine.

## Burtai coal mine overview and accident hazard analysis

### Burtai coal mine overview

The Burtai coal mine is a super-size mine located in Inner Mongolia, China, with an area of 192.8559 km^2^ and an average elevation of 1300 m. The Burtai coal mine has complex terrain, including many crisscrossing valleys. The geological reserve of its coal seam is 3.3 billion tons, and its recoverable reserves account for 1.85 billion tons. According to the annual mining yield estimation of 20 million tons, the service life of this mine will be 71.3 years. There are a total of ten recoverable coal seam layers in this mine. The currently mined layers are as follows: 2–2 in the first layer and 4–2 in the second layer. The coal seam dip angle is 1°–3°. The coal seam thickness is in the range of 0.87–7.32 m, with an average value of 3.78 m. The resource reserve utilization thickness is in the range of 0.87–7.24 m, with an average value of 3.67 m. The recoverable thickness in the bifurcation area is between 0.90 and 5.00 m, with an average value of 2.28 m. The recoverable thickness in the combined area is between 0.87 and 7.24 m, with an average value of 5.90 m. The thickness of the coal seam changes regularly with a tendency of thickening in the middle and thinning in the southeast and west, i.e., thickening in the combined area and thinning in the bifurcation area.

### Accident hazard analysis

Several mining approaches have been adopted in the Burtai coal mine, including inclined shaft, adit, and vertical shaft. Also, partition extraction ventilation is used in this mine. Initially, five shafts were arranged, including the main inclined shaft, an auxiliary adit, a Sundingholuo air intake shaft, an industrial square air return inclined shaft, and a Sundingholuo air return shaft. The first three shafts induct air, and the last two shafts return air. The total air volume of the mine is 15,212 m^3^/min; the total air intake and return volumes are 15,241 m^3^/min and 15,482 m^3^/min, respectively. Inverted ventilation for the entire mine is achieved by using multiple main fans with fan reversal. The coal mining method is comprehensive mechanized coal mining along the long wall. The roof is managed by using the total straddle method. Further, tunneling is achieved by using continuous shearers, anchor diggers, and comprehensive diggers. Finally, shuttle cars and conveyors are used to transport coal.

According to the gas grade certification of the Burtai coal mine in 2021, the relative gas emission is 0.88 m^3^/t, and the absolute gas emission is 23.99 m^3^/min. The relative and absolute emissions of carbon dioxide are 0.41 m^3^/t and 11.01 m^3^/min, respectively. The mine has a low gas level without outburst danger related to coal and gas. The coal dust explosion indices of the 2–2 coal and the 4–2 coal are 33.82% and 36.55%, respectively, which indicates high explosive properties. The spontaneous combustion tendency grades of the 2–2 coal seam and the 4–2 coal dust fall in class II spontaneous combustion, and the ignition period is usually 1–3 months. When there is floating coal in the roadway with poor ventilation, spontaneous combustion may occur. Moreover, a coal dust explosion can be caused by a gas explosion, electrical equipment detonation, friction sparks, impact sparks, a fire caused by electric welding, and spontaneous coal combustion.

Depending on the type of coal mined and the conditions of the working face, the fire may spread rapidly, intensify and burn for a long time. Because of this, the normal and safest choice is self-escape^29^. For miners, it is very difficult to find a reasonable and safe emergency escape route in such a complex underground environment.

Table 1 indicated four possible affecting factors based on pathfinding and communication problems of possible environmental conditions under which mine escaping can occur. The environmental conditions will detect the most appropriate technology to be used and various limitations on the escaping. For pathfinding and decision-making, what matters is visibility and words, which may not always be possible in the process of self escape^30^. Therefore, it is necessary to analyze and plan the emergency escape route in advance through numerical simulation, so as to provide guarantee and support for underground workers to make reasonable choices in emergency situations.

**Table 1:** Possible factors affecting escape.

Affecting factor 1	Affecting factor 2	Affecting factor 3	Affecting factor 4
No visibility	No speech	No Tactile	No sign signal communication

## Mathematical model of mine fire rescue algorithm

Based on the analysis on the potential hazard sources above and factors affecting the escape routes of Burtai coal mine, combined with the reality of Burtai coal mine, a fire emergency escape simulation algorithm for the coal mine is proposed and described as follows:

First, an average walking speed of a mine worker is calculated. The walking speed of a mine worker at a moment *t* is given by:

1 $$ v(t) = v_{0} \frac{{k_{t} }}{{k_{1} k_{2} k_{3} }}, $$

where *v*(*t*) is the walking speed of a worker at a moment *t* in m/s; *v*_0_ denotes the normal walking speed (m/s); *k*_*t*_ is the walking speed variation with time; *k*_1_ denotes the impact coefficient of a roadway slope, and 0 < *k*_1_ < 1 when the roadway slope is *J* > 0, *k*_1_ = 1 when *J* = 0, and *k*_1_ > 1 when *J* < 0; *k*_2_ represents the impact coefficient of roadway obstacles, and *k*_2_ = 1 when either there are no obstacles on a roadway or obstacles do not affect workers’ walking, and 0 < *k*_2_ < 1 when there are obstacles on a roadway and they affect workers’ walking; *k*_3_ is the impact coefficient of the roadway shape, and 0 < *k*_3_ < 1 when the roadway shape is unfavorable for walking; otherwise, *k*_3_ = 1.

The time spent by a worker passing through a roadway is given by:

2 $$ t(i) = \frac{l(i)}{{\overline{v}(i)}}, $$

where *l*(*i*) is the length of roadway *i* (m), and $\overline{v} (i)$ the average speed of the worker passing through the roadway *i* (m/s).

The time required by the smoke front to pass through a roadway *i* is obtained by:

3 $$ t_{f} (i) = \frac{l(i)}{{\overline{v}_{f} (i)}}, $$

where $\overline{v}_{f} (i)$ is an average velocity of the smoke front passing through the roadway *i* (m/s).

In a directed graph *G* = (*V*, *E*), *V* is a collection of nodes, and $V = \left\{ {v_{1} ,v_{2} , \cdots v_{m} } \right\}$; *m* denotes the number of nodes, and $m = \left| v \right|$; *E* is the collection of branches, and $E = \left\{ {e_{1} ,e_{2} , \cdots e_{n} } \right\}$; *n* is the number of branches, and $n = \left| E \right|$. In a directed graph *G*, *G*_*w*_ is the polluted area, and other sub-graphs represent safe areas and are denoted as *G*_*s*_^28^, showing the following relationship with the polluted area:

4 $$ G_{s} = G - G_{w} . $$

In the escape process, workers may follow the wind direction or run against the wind direction. Therefore, a depth-first search (DFS) method based on an undirected graph should be used to determine an escape route^31^. The collection of nodes from the ending node of the current branch to the pathways in a safe area is denoted by *P*_*s*_ and expressed as:

5 $$ P_{s} = \{ P_{i} |V^{ - } (P_{i} ) = v_{t} ,(v_{j} ,v_{t} ) = e_{f} ;V^{ + } (P_{i} ) = v_{k} ,v_{k} \in V(G_{s} )\} . $$

When a fire occurs, the front of the fire smoke flow may spread to a certain location $\xi$ in a branch $e_{k}$ through different paths. The set of these pathways is denoted by $P_{f} (e_{k} ,\xi )$ and expressed as follows:

6 $$ P_{f} (e_{k} ,\xi ) = \{ P_{i} |V^{ - } (P_{i} ) = v_{t} ,V^{ + } (P_{i} ) = \xi ,(v_{f} ,v_{t} ) = e_{f} \} . $$

The path $P_{fq} (e_{k} ,\xi )$ and time $t_{fq} (e_{k} ,\xi )$ of the front of the fire smoke flow that first spreads to a location $\xi$ in a branch $e_{k}$ are respectively given by:

7 $$ P_{fq} (e_{k} ,\xi ) = \{ P_{i} |t_{f} (P_{i} ) < t_{f} (P_{k} ),P_{i} \in P_{f} (\xi ),P_{k} \in P_{f} (e_{k} ,\xi )\} , $$

8 $$ t_{f} (e_{k} ,\xi ) = \frac{{x_{f} }}{{\overline{v}_{f} (k,x_{f} )}} + \sum\limits_{j = 0}^{f} {t_{f} (j)} , $$

9 $$ t_{fq} (e_{k} ,\xi ) = t(P_{fq} (e_{k} ,\xi )), $$

where $t_{f} (\xi )$ is the time needed for the front of the smoke flow to spread (s); $x_{f}$ is the residual distance of the smoke flow (m); $\overline{v}_{{_{f} }} (k,x)$ denotes an average spreading velocity of the front of the smoke flow at the residual distance $x_{f}$ of the current branch $e_{k}$ (m/s); $f$ is the number of branches before the branch $e_{k}$ on the smoke flow spreading path $P_{i} (P_{i} \in P_{f} )$; $t_{f} (j)$ represents the time needed for the smoke front to pass through a roadway $j$ (s).

An escape route is determined as a collection of escape pathways *P*_*esc*_ selected from the collection of safe pathways *P*_*s*,_ which can be expressed as follows:

10 $$ P_{esc} = \{ P_{i} |t_{r} (e_{k} ,\xi ) < t_{fq} (e_{k} ,\xi ),e_{k} \in P_{s} \} , $$

11 $$ t_{r} (e_{k} ,\xi ) = \frac{x}{{\overline{v}(k,x)}} + \sum\limits_{j = 0}^{r} {t(j)} , $$

where $t_{r} (e_{k} ,\xi )$ is the time needed for workers to reach a location $\xi$ in a branch $e_{k}$ (s); $t_{fq} (e_{k} ,\xi )$ is the shortest time needed for the smoke flow to reach the location $\xi$ (s); $e_{k}$ denotes a branch in the collections *P*_*s*_ and $P_{f} (e_{k} ,\xi )$; *x* is the remaining distance of escape (m); $\overline{v} (k,x)$ denotes an average walking speed of workers to reach the distance *x* of the current branch $e_{k}$ (m/s); *f* is the number of branches before branch $e_{k}$ on the current escape path $P_{i} (P_{i} \in P_{esc} )$; $t\left( j \right)$ the time needed for workers to pass through a roadway *j* (s).

The best escape route is the shortest path in the collection of all feasible escape paths from the fire location to any node in the safe areas, and it is defined as follows:

12 $$ P_{opt} = \{ P_{i} |t(P_{i} ) < t(P_{k} ),P_{i} \in P_{esc} ,P_{k} \in P_{esc} \} , $$

13 $$ t(P_{i} ) = \sum\limits_{j}^{{\left| {P_{i} } \right|}} {t(e_{j} )} \;\;\;(e_{j} \in P_{i} ), $$

where $t(P_{i} )$ is the time needed for workers to pass $P_{i} (s)$, and $\left| {P_{i} } \right|$ is the scale of $P_{i}$, i.e., the number of branches in $P_{i}$.

**Figure 1:**
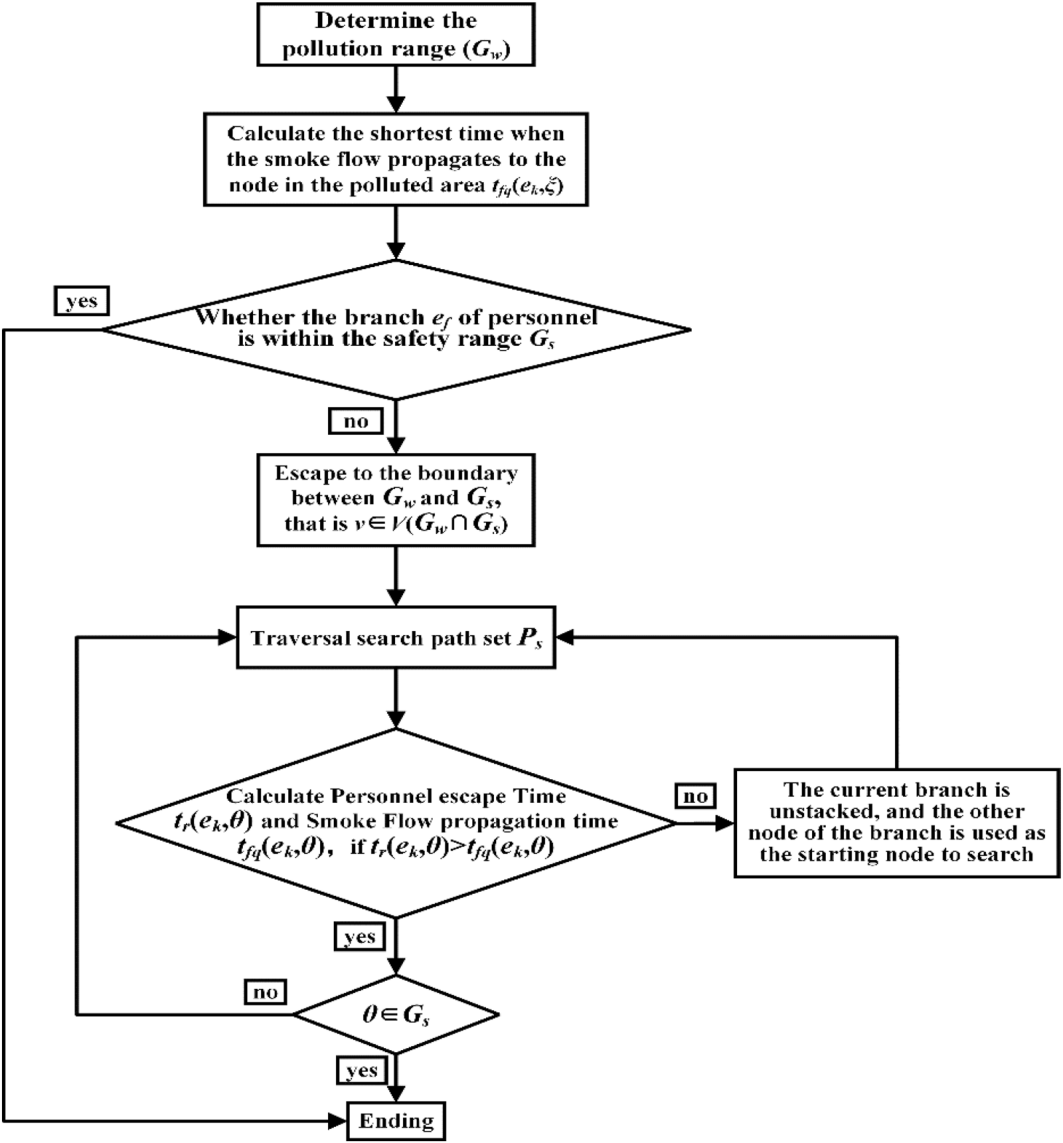
Numerical calculation process.

Based on the established mathematical model, the simulations are conducted using MATLAB software. First, the simulation calculation program was written, and then the simulation calculations were performed, as shown in Fig. 1.

## Analysis of simulation results

**Figure 2:**
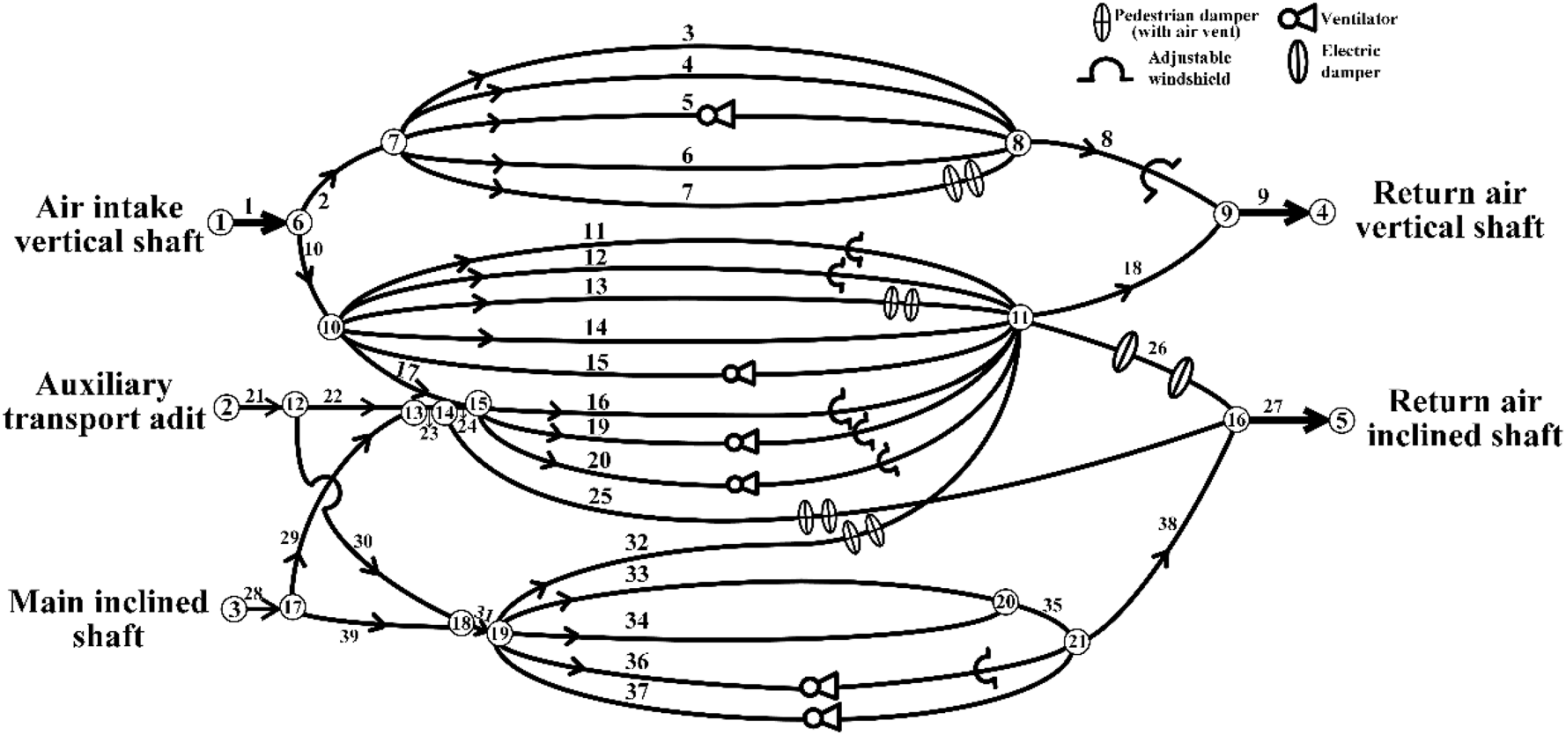
Ventilation network diagram of the Burtai coal mine.

A ventilation network diagram was drawn according to the overall structure of the Burtai coal mine, as shown in Fig. 2. A ventilation network topology of the Burtai coal mine was constructed based on the diagram displayed in Fig. 2, as shown in Table 2.

**Table 2:** Topological relation of ventilation network in Buertai Coal Mine.

Branch	Starting node	Ending node	Branch	Starting node	Ending node	Branch	Starting node	Ending node
1	1	6	14	10	11	27	16	5
2	6	7	15	10	11	28	3	17
3	7	8	16	15	11	29	17	13
4	7	8	17	10	15	30	12	18
5	7	8	18	11	9	31	18	19
6	7	8	19	15	11	32	19	11
7	7	8	20	15	11	33	19	20
8	8	9	21	2	12	34	19	20
9	9	4	22	12	13	35	20	21
10	6	10	23	13	14	36	19	21
11	10	11	24	14	15	37	19	21
12	10	11	25	14	16	38	21	16
13	10	11	26	11	16	39	17	18

**Figure 3:**
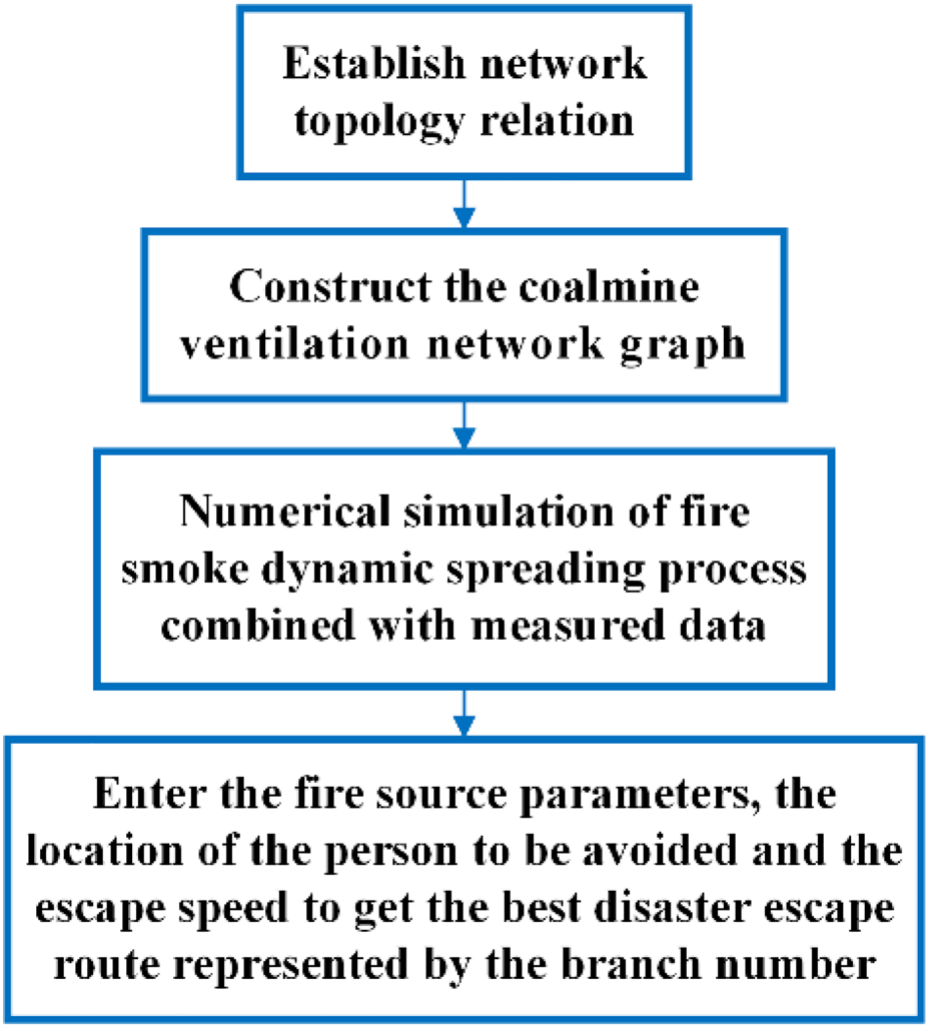
Modeling process of the best disaster escape route.

Based on the monthly ventilation report of the Burtai coal mine, the simulation data of the dynamic spreading process of the smoke flow in a fire were obtained, as shown in Table 3. Accurate construction of ventilation network extension and supplement relationship is crucial to the establishment of a ventilation network graph because it defines the correctness of the ventilation network graph. The ventilation network graph reflects the branches and circuits of the whole mine and is the basis for determining a disaster escape route^32^. Based on the mathematical model of the mine fire spreading process and actual ventilation network data, the dynamic calculation of mine fire spread was conducted. According to the simulation results, fire source parameters, and personnel location, the main disaster escape routes were obtained. The main steps to determine an optimal escape route by numerical simulation are shown in Fig. 3.

**Table 3:** Simulation data of dynamic spreading process of fire smoke in Buertai Coal Mine.

Branch	Starting node	Ending node	Branch length/m	Flow rate/m^3^·s^-1^	Truncation area/m^2^	Slope	Average density/kg·m^3^	Perimeter/m
1	1	6	500	112	15	0.7	1.102	13
2	6	7	50	25	12	0.3	1.102	10
3	7	8	120	6	10	0.3	1.104	9
4	7	8	120	7	10	0.1	1.105	9
5	7	8	120	5	10	0.2	1.102	9
6	7	8	120	3	10	0.1	1.103	9
7	7	8	120	4	10	0.1	1.105	9
8	8	9	300	25	12	0.2	1.121	10
9	9	5	367	116	15	0.1	1.161	13
10	6	10	65	87	12	0.8	1.101	10
11	10	11	160	6	10	0.1	1.102	9
12	10	11	160	54	10	0.2	1.102	9
13	10	11	160	4	10	0.3	1.103	9
14	10	11	160	9	10	0.1	1.102	9
15	10	11	160	9	10	0.1	1.103	9
16	15	11	160	5	10	0.2	1.105	9
17	10	15	38	5	12	0.3	1.101	10
18	11	9	78	91	12	0.2	1.121	10
19	15	11	129	5	10	0.1	1.120	9
20	15	11	126	5	10	0.1	1.110	9
21	1	12	460	28	15	0.8	1.101	13
22	12	13	34	10	12	0.1	1.102	10
23	13	14	38	14	12	0.1	1.102	10
24	14	15	32	10	12	0.2	1.103	10
25	14	16	340	4	10	0.3	1.112	9
26	11	16	45	10	12	0.1	1.138	10
27	16	5	378	15	0.7	0.7	1.140	13
28	1	17	656	34	15	0.6	1.101	13
29	17	13	56	4	10	0.1	1.101	9
30	12	18	129	18	10	0.1	1.101	9
31	18	19	45	48	10	0.1	1.102	9
32	19	11	345	4	10	0.1	1.105	9
33	19	20	89	10	10	0.1	1.106	9
34	19	20	89	23	10	0.1	1.108	9
35	20	21	67	33	10	0.1	1.109	9
36	19	21	235	6	10	0.1	1.110	9
37	19	21	235	5	10	0.1	1.120	9
38	21	16	68	44	12	0.1	1.136	10
39	17	18	18	30	12	0.2	1.104	10

**Figure 4:**
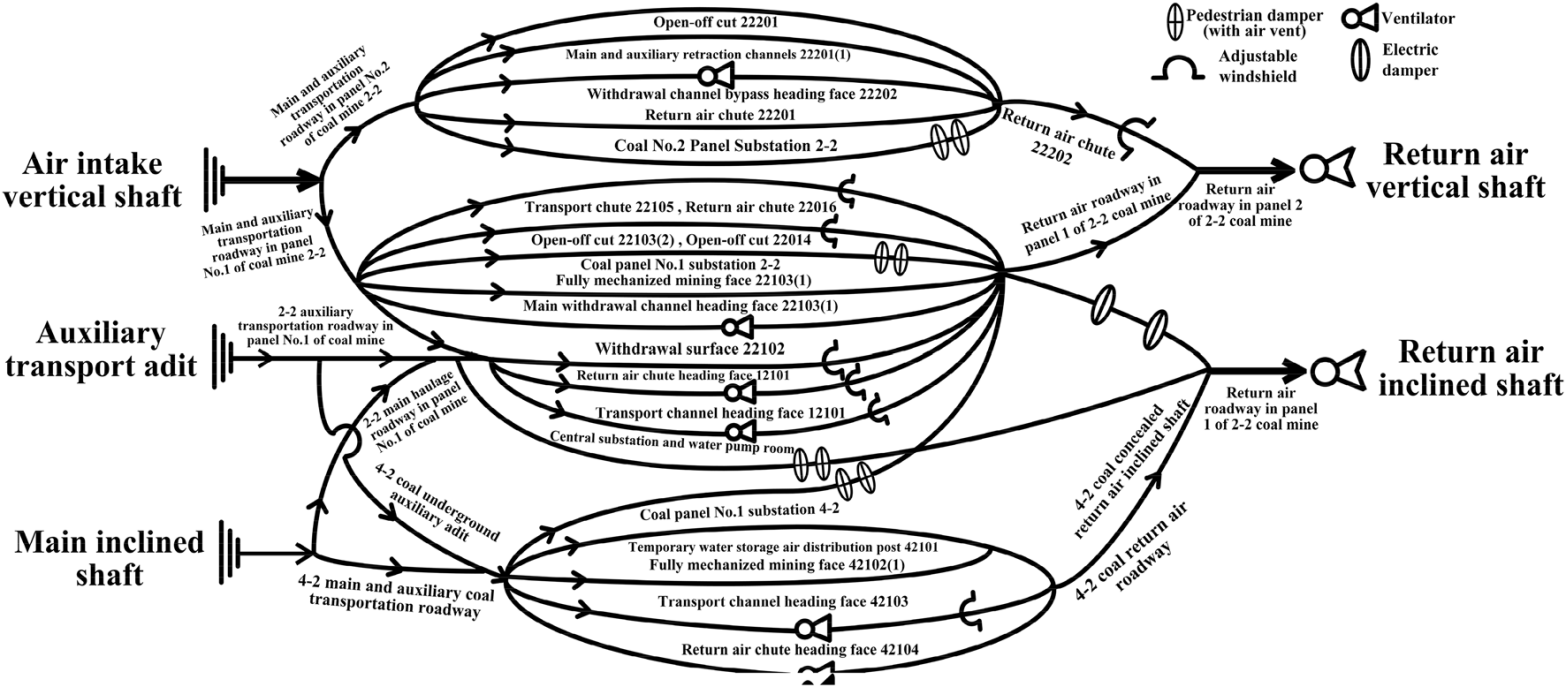
Schematic diagram of the ventilation network of the Burtai coal mine.

The ventilation network diagram of the Burtai coal mine is shown in Fig. 4. According to the numerical simulation results and Fig. 4, the main escape routes can be determined for disasters that occur at the coal mining working face and the tunneling working face, as well as in the main inclined shaft, auxiliary adit, and the main air intake roadway.

There are three main disaster escape routes at the coal mining working face, which are as follows (All escape routes below can be found in Fig. 4):

**Route 1:** 22,103 (1) comprehensive mining face → 22,103 transport chute → 2–2 auxiliary coal transport roadway → auxiliary transport adit → ground;

**Route 2:** 22,103 (2) comprehensive mining face → 22,104 transport chute → 2–2 auxiliary coal transport roadway → auxiliary transport adit → ground;

**Route 3:** 42,102 comprehensive mining face → 42,102 transport chute → 4–2 auxiliary coal transport roadway → 4–2 dark auxiliary coal transport adit → 2–2 auxiliary coal transport roadway → auxiliary transport adit → ground.

There are six main disaster escape routes at the tunneling working face, which are as follows:

**Route 1:** 12,101 transport chute tunneling face → 12 auxiliary coal adit → 2–2 auxiliary coal transport roadway → auxiliary transport adit → ground;

**Route 2:** 12,101 return air chute tunneling face → 12 auxiliary coal adit → 2–2 auxiliary coal transport roadway → auxiliary transport adit → ground;

**Route 3:** 22,201 return air chute tunneling face → central auxiliary transport roadway → 2–2 coal auxiliary transport roadway → auxiliary transport adit → ground;

**Route 4:** 22,202 retreat chute tunneling face → central auxiliary transport roadway → 2–2 coal auxiliary transportation roadway → auxiliary adit → ground;

**Route 5:** 42,103 transport chute tunneling face → 42,103 transport chute → 4–2 auxiliary coal transport roadway → 4–2 dark auxiliary coal transport adit → 2–2 auxiliary coal transport roadway → auxiliary transport adit → ground;

**Route 6:** 42,104 return air chute tunneling face → 42,103 transportation chute → 4–2 auxiliary coal transport roadway → 4–2 dark auxiliary coal transport adit → 2–2 auxiliary coal transport roadway → auxiliary transport adit → ground.

There are four main disaster escape routes from the fire with the reversed wind in the main inclined shaft, the auxiliary transport adit, and the main air intake roadway, which are as follows:

**Route 1**: staff in the 2–2 coal and panel 1 → 2–2 coal return air roadway → industrial square return air inclined shaft → ground;

**Route 2:** staff in the 4–2 coal → 4–2 coal return air roadway → 4–2 dark coal return air inclined shaft → 2–2 coal return air roadway → industrial square return air inclined shaft → ground;

**Route 3:** staff in the 2–2 coal and panel 2 → central auxiliary transport roadway → 32# connected roadway grid bypass → central return air roadway to wait for rescue;

**Route 4:** staff at the bottom of the air intake vertical shaft → central auxiliary transport roadway → 32# connected roadway grid bypass → central return air roadway to wait for rescue.

## Conclusions

Based on a self-established mathematical model of a mine fire disaster relief algorithm, numerical simulations of a dynamic spreading process of the fire smoke are conducted, and the main disaster escape route in the Burtai coal mine is determined. The main conclusions of this study can be drawn as follows:

1. Potential accidents and hidden danger of the coal mine are identified based on the geographical location, geological conditions, and actual production situations of the Burtai coal mine.
2. A mathematical model of a mine fire rescue algorithm is constructed for the hidden danger and potential accidents. This model is used to conduct numerical simulation on the dynamic spreading process of the fire smoke flow in the Burtai coal mine.
3. Several escape routes are determined based on the simulation results, including the main escape route at the coal mining working face, the main escape route at the tunneling working face, and the main escape route against the reverse wind in the main inclined shaft, auxiliary adit, and the main air intake roadway.

In future work, the main disaster escape routes in all mining areas of the Burtai coal mine could be determined, and technical support for improving the emergency rescue plan of the coal mine could be provided.
